# Genetic Diversity of *Coxiella burnetii* in Iran by Multi-Spacer Sequence Typing

**DOI:** 10.3390/pathogens11101175

**Published:** 2022-10-11

**Authors:** Ashraf Mohabati Mobarez, Neda Baseri, Mohammad Khalili, Ehsan Mostafavi, John Stenos, Saber Esmaeili

**Affiliations:** 1 Department of Bacteriology, Faculty of Medical Sciences, Tarbiat Modares University, Tehran 1411713116, Iran; 2 Department of Epidemiology and Biostatics, Research Centre for Emerging and Reemerging Infectious Diseases, Pasteur Institute of Iran, Tehran 1316943551, Iran; 3 National Reference Laboratory for Plague, Tularemia and Q Fever, Research Centre for Emerging and Reemerging Infectious Diseases, Pasteur Institute of Iran, Akanlu, KabudarAhang, Hamadan 6556153145, Iran; 4 Department of Pathobiology, Faculty of Veterinary Medicine, Shahid Bahonar University of Kerman, Kerman 7616913439, Iran; 5 Australian Rickettsial Reference Laboratory, University Hospital Geelong, Geelong, VIC 3220, Australia

**Keywords:** *Coxiella burnetii*, Q fever, Genotyping, MST, Iran

## Abstract

*Coxiella burnetii*, the zoonotic agent of Q fever, has a worldwide distribution including Iran. However, no information regarding the circulating genotype of this infection has been reported in Iran. This study aimed to evaluate the genetic diversity of *C. burnetii* in Iran using the multi-spacer sequence typing (MST) method. First, 14 positive *C. burnetii* samples (collected from four sheep, three goats, and seven cattle) were confirmed using quantitative polymerase chain reaction (qPCR) targeting the *IS1111* gene. Then, ten spacers (Cox 2, 5, 18, 20, 22, 37, 51, 56, 57, and 61) were amplified using PCR for future MST analysis. The *in-silico* MST genotyping analysis of domestic ruminant samples revealed two new alleles (Cox5.11 and Cox56.15) in Cox5 and Cox56 loci that led to the emergence of four novel MST genotypes (MST62, 63, 64, and 65) and one MST genotype that has been previously described (MST61). This study showed the circulation of five MST *C. burnetii* genotypes among Iranian domestic ruminants. Understanding the *C. burnetii* genotypic profiles is critical in determining and preventing Q fever outbreaks.

## 1. Introduction

Q fever is a very important zoonotic disease worldwide. The infection is caused by an intracellular bacterium called *Coxiella burnetii* [1]. The global distribution, reservoirs, routes of transmission, and vectors of *C. burnetii* are very diverse. Therefore, the epidemiological features of Q fever are very complex [1,2]. Genotyping studies of *C. burnetii* isolates provide helpful information on types of circulating strains at the farm, regional, and country levels. In addition, molecular epidemiology can be used to identify the source of infection in humans and animals, the circulating and endemic genotypes, and virulent strains in acute and chronic Q fever. Finally, these findings help to establish preventive and control measures [3]. 

Since *C. burnetii* is highly infectious, its culture and purification are very dangerous for laboratory staff and require access to biosafety level 3 (BSL3) laboratory conditions. In addition, the culture of this agent requires enriched mediums and animal-derived cell culture systems [4]. Due to these factors, it is impossible to cultivate *C. burnetii* in the laboratories of some countries. Therefore, there are no comprehensive comparative studies on this bacterium in these countries [1,5]. On the other hand, differentiation between diverse *C. burnetii* strains has not been successful using serological methods [2]. 

Access to molecular methods and genome sequencing has led to great advances in bacterial typing. These techniques allow **scientists to** direct comparisons and evolutionary relationships between different strains using bioinformatics. Whole-genome sequencing is the ultimate method for direct comparison between strains. However, these methods are not accessible in many laboratories because there are limited financial resources in many countries. On the other hand, differentiation of *C. burnetii* strains based on sequencing of 16SrDNA, *com1*, *mucZ*, and *isocitrate dehydrogenase* genes does not provide enough information because of the similarity (more than 99% homologous) between different strains in the sequence analysis [2,6]. Therefore, these methods cannot be used to distinguish between different *C. burnetii* strains [2,7]. Since 2005, highly differentiated methods have been developed for *C. burnetii* genotyping, including multi-spacer sequence typing (MST), multiple-locus variable-number tandem repeat analysis (MLVA), and single nucleotide polymorphism (SNP) [2,7]. MST is based on intergenic region sequencing, and these regions are potentially variable since they are subject to lower selection pressure than the adjacent genes. For *C. burnetii* genotyping, Glazunova et al. selected ten spacers that had the most variations. This technique was able to classify 173 isolates of *C. burnetii* into 30 genotypes [8]. Due to its high-resolution power, this technique allows direct comparison of results between different laboratories. Another advantage of this technique is the ability to detect new alleles and changes within the studied spacers [1].

Since Q fever is an endemic zoonotic disease in Iran, there are many studies on the prevalence of *C. burnetii* [9,10,11,12]. However, there are no studies on the genotyping of this bacterium in livestock and humans. Various molecular studies in milk and animal abortion samples show the high prevalence of this bacterium in different parts of Iran. The *C. burnetii* genotyping data helps healthcare systems to explore Q fever epidemiology and putative outbreak control criteria. Therefore, this study aimed to genotype *C. burnetii* strains identified in Iran using the MST method.

## 2. Materials and Methods

### 2.1. Samples

**Figure 1:**
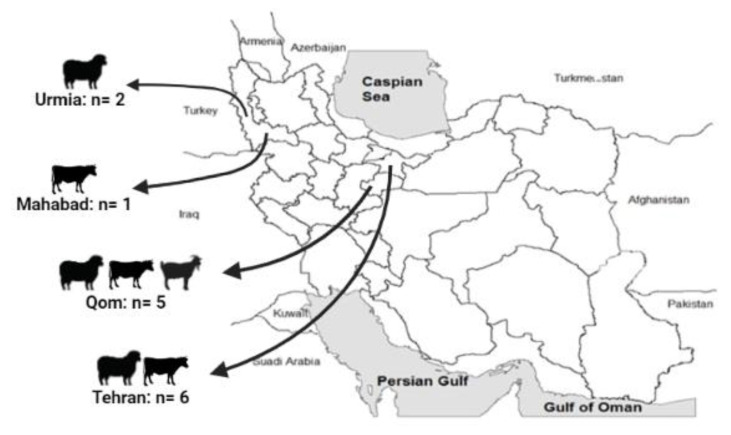
Geographic distribution of selected samples for *Coxiella burnetii* genotyping in Iran in the present study. Samples were obtained from 2 sheep in Urmia, 1 goat in Mahabad, 2 sheep, 1 goat, and 2 cattle in Qom, and 4 cattle, 1 goat, and 1 sheep in Tehran.

In this study, we used various human and animal samples available in our biobank. These samples were collected during our previous studies [10,13,14,15] and were positive for *C. burnetii*. Seven animal abortion samples (two sheep, two goats, and three cattle) and seven milk samples (two sheep, one goat, and four cattle) were selected for MST genotyping (Table 1 and Figure 1). None of the human specimens had a sufficient level of *C. burnetii* DNA to be used in this study.

### 2.2. DNA Extraction and Quantitative Polymerase Chain Reaction (qPCR)

Using the Roche High Pure PCR Template Preparation Kit (Roche, Germany), DNA was extracted from 50 mg of aborted tissue and spleen samples or 200 μL of abortion fluids and milk samples, according to the manufacturer’s instructions.

For quantitative polymerase chain reaction (qPCR), we used DNA of *C. burnetii* Nine Mile, phase I strain (RSA 493), as a positive standard control, which was phenol-killed, purified, and lyophilized (Institute of Virology, Slovak Academy of Sciences, Bratislava, Slovak Republic). Based on the measured DNA concentration (50 ng/μl), the length of the published sequence of the *C. burnetii* Nine Mile (RSA 493) genome (1,995,275 bp), and also the mass of each *C. burnetii* genome (2.3 × 10^-7^ ng), the theoretical number of genome equivalents (GE) was calculated to be 2.24 × 108 GE per μl. Each *C. burnetii* Nine Mile strain (RSA 493) genome contains 20 copies of the *IS 1111* gene. Therefore, the concentration of the *IS1111* gene was 4.48 × 109 per μl in our standard control [16]. Ten consecutive dilutions (10-folds) were used for the standard control stock standard curve. Quantification of the approximate bacterial load and the quantification cycle (Cq) values were achieved using this standard. Finally, all samples were tested against the standard curve and Ct values for all samples were determined. Samples with Ct values of 31 or lower in qPCR were selected for MST genotyping. Furthermore, animal species, sample source, and origin were considered in the selection of the samples tested.

### 2.3. Multi-Spacer Sequence Typing (MST)

MST analyses of *C. burnetii* were performed on selected samples using ten spacers (Cox 2, 5, 18, 20, 22, 37, 51, 56, 57, and 61) (Table 2). PCR conditions and primer sequences have been previously described [8]. Briefly, each amplification reaction contained 12.5 μL of TEMPase Hot Start 2x Master Mix BLUE (Ampliqon, Odense, Denmark), 0.5 μM of each primer, 5 μL of DNA sample (in the final concentration of 5 ng), and 5.5 µL of double-distilled water in a final reaction volume of 25 µL. The PCR product for each gene was electrophoresed on 1% agarose gel to ensure the presence of the desired band and the absence of nonspecific bands. PCR products were sequenced by ABI 3500 Genetic Analyzer (Applied Biosystems, Foster City, CA, USA) using forward and reverse primers. Sequencing results were initially reviewed using Chromas software (http://www.technelysium.com.au/chromas.html). In addition, sequencing data were edited using SeaView v.4 software (http://pbil.univ-lyon1.fr/software/seaview accessed on 5 January 2018). Allele numbers and MST genotype were determined by comparison with the MST database of *C. burnetii* (https://ifr48.timone.univ-mrs.fr/mst/coxiella_burnetii/ accessed on 20 February 2018).

Sequences that were not identical to the alleles in the database were considered novel alleles. These novel alleles were submitted to the MST database of *C. burnetii*. After examining the sequence of these alleles for each spacer against the database, these sequences were identified as new alleles. Also, profiles that did not resemble database data were considered a new MST genotype of *C. burnetii* and all identified MST genotypes in this study were recorded in the database.

### 2.4. Data Analysis

Data of all registered MST genotype profiles were extracted from the MST database and publications. All sequences of MST genotypes were aligned and used for phylogenetic analysis using the MEGA X version 10.2.2 software. The evolutionary distances were inferred using the neighbor-joining method (computing the maximum composite likelihood method) and expressed in the number of base substitutions per site by pair-wise comparison of 48 nucleotide sequences.

Also, the minimum-spanning tree was built using the goeBURST full algorithm (PHYLOViZ 2.1) for ten allelic profiles of all STs.

## 3. Results

In this study, a novel allele for Cox5 spacer was observed in two samples (AG1 and AS3). One mutation had occurred in nucleotides position 133 [Guanine (G) to Thymine (T) mutation] in the sequence of this novel allele compared with allele Cox5.4. This novel allele of Cox5 was recognized by the MST database as a new allele (Cox5.11). Moreover, a novel allele for the Cox56 spacer was observed in one sample (MB14), in which four mutations along with two deletions of nucleotides have occurred in the sequence of this novel allele relative to Cox56.10. This novel allele of Cox56 was recognized by the MST database as Cox56.15.

The discriminatory power of MST genotyping was calculated using Simpson’s diversity index (DI), which was 0.758. The Cox2, Cox22, and Cox57 spacers had only one allele in our samples. The Cox56 spacer had four different alleles with the highest discriminatory power (0.626) among all spacers. 

Based on the comparison of our data with MST data bank, we found five MST genotypes (MST61-65) in our samples. Among these MST genotypes, four MST genotypes (MST62-65) were novel (Table 3). MST61, which was previously reported in Polish dairy cattle, was found in cattle milk from Tehran County (Capital of Iran). In this study, MST62 was the most prevalent (42.8%) MST genotype of *C. burnetii*. This genotype was found in six samples, including cattle milk (Tehran County), one cattle abortion (Tehran County), one goat milk (Qom County, central Iran), one goat abortion (Mahabad county, northwestern Iran), and two sheep milk (Qom County) samples. In addition, MST63 was found in two samples (one goat abortion and one sheep abortion originating from Tehran County). MST63 was closely related to MST62 (nine spacers), but this MST genotype was different from one spacer (Cox5) compared with MST62. MST64 was the second most prevalent (28.6%) MST genotype of *C. burnetii* among our samples, which was found in four samples, including in cattle milk (Qom County), one cattle abortion (Tehran County), and two abortion (Urmia County, northwest of Iran) samples. MST64 was different in only one spacer (Cox56) compared with MST62. Finally, MST65 was found in a cattle milk sample (Qom County), and this genotype was different in the Cox56 spacer compared with MST62 and MST63.

**Figure 2:**
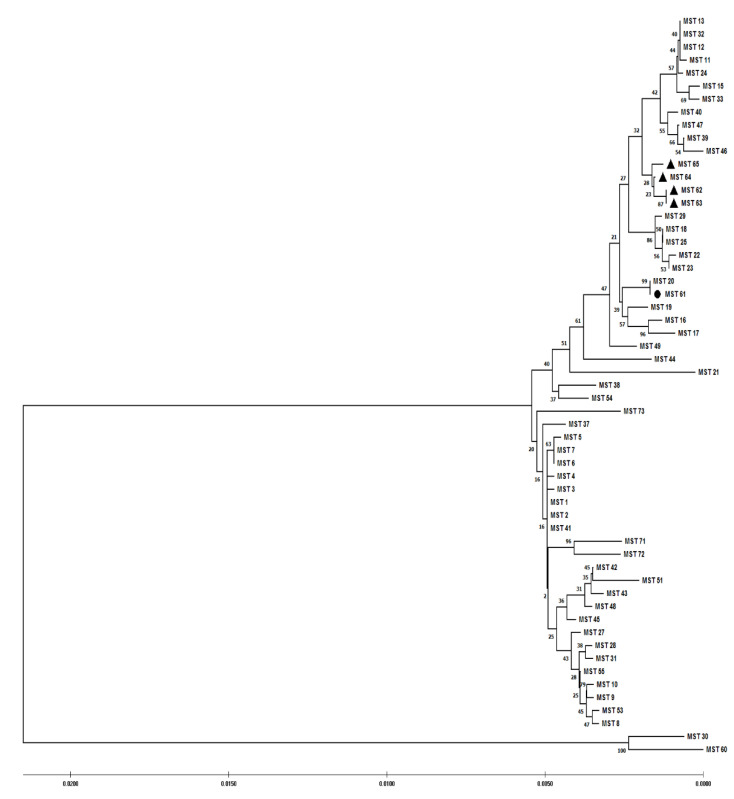
Neighbor-joining algorithm analysis of *Coxiella burnetii* multi-spacer sequence typing (MST) groups based on the connecting of 10 loci using computing the maximum composite likelihood method by Mega software. MSTs 61-65 are Iranian *C. burnetii* genotypes. MSTs 62-65 were our novel genotypes. MST61 was previously reported in Poland and Belgium. The symbol ▲ indicates novel detected genotypes in the present study (MST62-MST65), whereas the symbol ● indicates a previously reported genotype (MST61) that was also observed in the present study.

**Figure 3:**
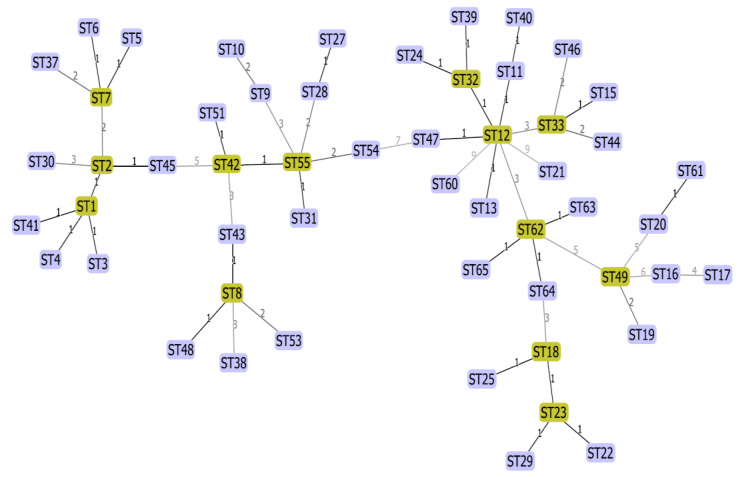
Multi-spacer sequence typing (MST) analysis using the minimum spanning tree algorithm for extracted data from the *Coxiella burnetii* database.

**Figure 4:**
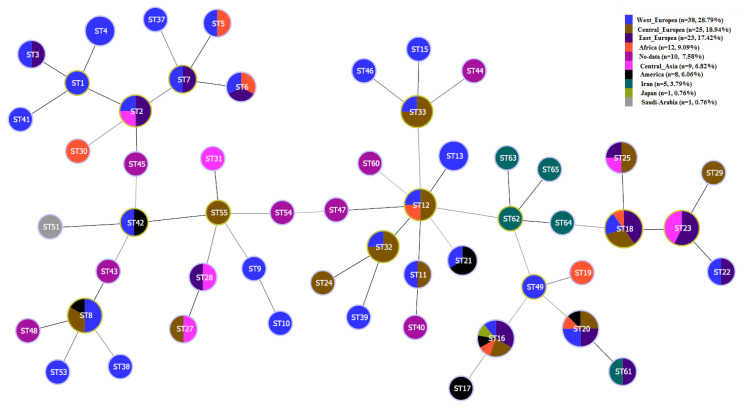
Analysis of multi-spacer sequence typing (MST) results using the minimum spanning tree algorithm for extracted data from *Coxiella burnetii* database based on the strain origin.

**Figure 5:**
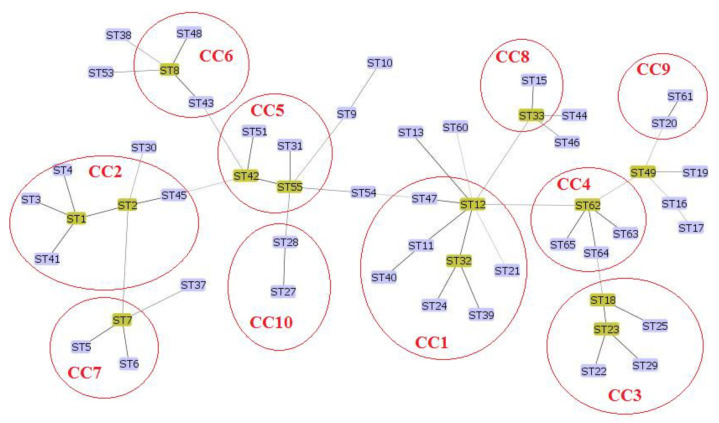
Clustering analysis of *Coxiella burnetii* in the minimum spanning tree algorithm based on the maximum difference in one allele using multi-spacer sequence typing (MST). It created ten clonal complexes (CC1-10) and fifteen singletons (MST38, MST53, MST30, MST37, MST9, MST10, MST54, MST13, MST60, MST44, MST46, MST49, MST16, MST17, and MST19).

**Figure 6:**
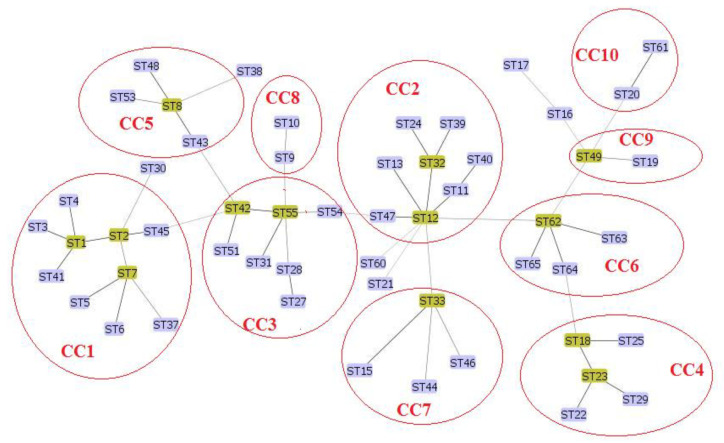
Clustering analysis of *Coxiella burnetii* in the minimum spanning tree algorithm based on the difference between the two alleles in multi-spacer sequence typing (MST). It emerged ten clonal complexes (CC1-10) and six singletons (MST16, MST17, MST21, MST60, MST38, and MST30).

Analysis of our data with available data of MST genotyping of *C. burnetii* using the maximum composite likelihood method (Mega software) showed (Figure 2) that our novel genotypes (MST62-65) were very closely related to each other. Also, MST61 was closely related to MST20. Furthermore, the minimum spanning trees algorithm was performed on these data by extracting the data deposited in the MST database for *C. burnetii* and combining those data with the obtained data from the present study. MSTs analysis revealed that the new Iranian genotypes of *C. burnetii* (MST62-65) complement the phylogenetic tree of *C. burnetii*. The MST62 genotype was also identified as a node in the minimum spanning tree algorithm (Figure 3 and Figure 4). In clustering performed in this typing method based on the maximum difference in one allele, ten clonal (CC1-CC10) complexes and 15 singletons (MST38, MST53, MST30, MST37, MST9, MST10, MST54, MST13, MST60, MST44, MST46, MST49, MST16, MST17, and MST19) were created. Iranian samples were located in clonal complexes No. 4 (MST62-65) and No. 9 (MST61) (Figure 5). Moreover, in clustering performed based on the maximum difference between the two alleles, ten clonal complexes (CC1-CC10) and six singletons (MST16, MST17, MST21, MST60, MST38, and MST30) were created. Accordingly, Iranian samples were located in clonal complexes No. 6 (MST62-65) and No. 10 (MST61) (Figure 6).

## 4. Discussion

In the present study, the genetic diversity of *C. burnetii* strains in Iran was evaluated using the MST method and showed five MST genotypes in domestic animals including four novel MST genotypes. Domestic ruminants are the main source of Q fever infection in humans [17]. Therefore, it is important to understand *C. burnetii* prevalence and its genotypes among domestic ruminants to comprehend the epidemiological routes of infection. The MST method is a useful tool for investigating the genotypic variants of *C. burnetii* [8].

In the present study, 14 different samples of *C. burnetii* were selected (from domestic ruminants and their products in four cities) for genotyping by ten gene targets using the MST method. After analyzing the data, two new alleles were identified in Cox5 (Cox5.11 in two specimens) and Cox56 (Cox56.15 in one specimen) loci. In agreement with the present studies, Cox56 has been previously reported as highly polymorphic and variable loci that could represent new genotypes [18]. According to the new alleles in the present study, investigated samples were classified into five *C. burnetii* genotypes. Among identified genotypes in the present study, one genotype (MST61) has been previously described among *C. burnetii* strains originating from small ruminants in different parts of the world [18,19,20]. The MST61 genotype was reported for the first time in 2019 in dairy products (raw milk and cheese) obtained from cattle in Poland [20]. In the present study, MST61 was also identified in cattle milk in Tehran. In Belgium, MST61 was found in cattle abortion material, cattle stomach content, and goat milk [19]. In phylogenetic tree analysis based on *C. burnetii* MST typing, genotypes of Brazilian strains from goats (vaginal swab), cattle (fetus), and sheep (vaginal swab) appeared to have a close MST clone to MST61 and MST20 [18]. In MST analysis, high similarity and the common ancestor are observed between MST61 and MST20 [18,20]. Here, MST61 and MST20 are located in the same clonal complex (Figure 5 and Figure 6) in clustering analysis based on the difference between the alleles **in** MST genotyping. MST20 is a dominant genotype in many countries of Europe, Asia, Africa, and North America, especially in milk cattle [20,21]. However, in addition to livestock, MST20 has been identified in humans with Q fever and particularly in chronic Q fever cases from France [8]. These epidemiological studies displayed that the sequence type MST61 is predominately found in cattle but may also be transmitted to other domestic ruminants. Moreover, it should be considered a potential virulent genotype for human infections, possibly due to its strong correlation with MST20. More global studies are needed to improve these hypotheses. 

Except for MST61, the other four identified genotypes (MST62-65) in the present study have not yet been reported elsewhere in the world. Therefore, there is no data about their virulence, and further research is suggested to investigate the possible pathogenesis of these genotypes in humans and livestock. MST62 was identified in milk and abortion samples of cattle and goats as well as sheep milk. The MST analysis showed a close relation between MST62 and MST12, MST18, and MST49. The MST12 has been identified in Europe and Africa from various specimens, including cattle milk (in Algeria) [22], cheese produced from the milk of sheep, goats, and cattle (in Italy) [23,24,25], and human clinical samples (in Switzerland, France, and Senegal) [22,24,25]. The MST18 was isolated from human, goat, sheep, cattle, and tick specimens in the Horn of Africa (Ethiopia) and Europe (France, Spain, Italy, Romania, Slovakia, Germany, and Poland) [24]. In agreement with other studies [26,27], MST49 is closely related to MST19 (CC9) and can be considered an ancestor of the MST20 genotype (Figure 5). However, it did not cluster with MST20.

Using the minimum spanning tree algorithm, ST62 was considered a putative ancestral genotype (Figure 3) for MST63, MST64, and MST65, which were classified in the same clonal complex (CC6; Figure 6). 

In our study, MST63 had only been observed in abortion (goats and sheep) samples. In the cattle (milk and abortion) and sheep (abortion) samples, MST64 was also identified. The MST65 genotype was only detected in cattle milk. Although some samples (MB14 and MB47; AC3 and AC11; MB92 and MB98) belonged to the same host, source, and region, MST presented a distinct genotype for these specimens. For instance, samples of MB14 and MB47 were isolated from cattle milk in Qom County but classified into two different MST genotypes, including MST65 and MST64, respectively. This indicates the high genetic diversity of *C. burnetii* strains in Iran. In agreement with the present study, most studies worldwide have indicated various *C. burnetii* genotypes using MST. For instance, among 12 samples: (cattle: *n* = 6; sheep: *n* = 5; and human: *n* = 1) in Hungary, three MST genotypes of *C. burnetii* were identified [28]. Five MST patterns emerged from the analysis of *C. burnetii* among 20 biological samples collected from domestic ruminant farms in Italy [25]. In Asia, limited studies on the *C. burnetii* genotype have been performed. The MST51 genotype was reported in Saudi Arabia in an endocarditis patient [29]. In Asia, as elsewhere in the world, more genotypic studies are needed to determine the geographical distribution of *C. burnetii* genotypes among humans and livestock infections.

## 5. Conclusions

This study showed the variability of *C. burnetii* genotypes among domestic ruminants in Iran using MST. Comparisons of *C. burnetii* genotypes to the deposited data in the MST database showed that the circulating strains of *C. burnetii* in Iran were significantly different from those in other parts of the world. Two new alleles (Cox56.15 and Cox5.11) and novel MST genotypes (MST62-65) were reported during this study. In addition, previously described ST61 was reported in one sample. Understanding *C. burnetii* genotyping provides valuable information about the epidemiology of this bacterium in Iran and may help control outbreaks. Therefore, comprehensive studies on genotyping of this bacterium in Iran are recommended. In addition, further research is needed to determine the pathogenicity of novel MST genotypes in humans and livestock.

## Figures and Tables

**Table 1:** Data of selected samples for *Coxiella burnetii* genotyping.

Sample ID	Host	Source	Region (County)	Cq *
MB14	Cattle	Milk	Qom	27.32
MB47	Cattle	Milk	Qom	25.24
MB92	Cattle	Milk	Tehran	29.61
MB98	Cattle	Milk	Tehran	27.25
AC3	Cattle	Aborted Fetus Spleen	Tehran	29.02
AC11	Cattle	Aborted Fetus Spleen	Tehran	27.82
MG101	Goat	Milk	Qom	11.6
AG1	Goat	Aborted Fetal Fluids	Tehran	17.98
AG4	Goat	Aborted Fetal Fluids	Mahabad	31.07
MS45	Sheep	Milk	Qom	25.01
MS51	Sheep	Milk	Qom	25.46
AS3	Sheep	Aborted Fetal Fluids	Tehran	25.5
AS25	Sheep	Aborted Fetus Spleen	Urmia	25.91
AS26	Sheep	Aborted Fetus Spleen	Urmia	26.13

* Quantification cycle.

**Table 2:** Oligonucleotide sequence of primers used for multi-spacer sequence typing (MST).

Spacer	Forward Primer (5´–3´)	Reverse Primer (5´–3´)
Cox2	CAACCCTGAATACCCAAGGA	GAAGCTTCTGATAGGCGGGA
Cox5	CAGGAGCAAGCTTGAATGCG	TGGTATGACAACCCGTCATG
Cox18	CGCAGACGAATTAGCCAATC	TTCGATGATCCGATGGCCTT
Cox20	GATATTTATCAGCGTCAAAGCAA	TCTATTATTGCAATGCAAGTGG
Cox22	GGGAATAAGAGAGTTAGCTCA	CGCAAATTTCGGCACAGACC
Cox37	GGCTTGTCTGGTGTAACTGT	ATTCCGGGACCTTCGTTAAC
Cox51	TAACGCCCGAGAGCTCAGAA	GCGAGAACCGAATTGCTATC
Cox56	CAAGCTCTCTGTGCCCAAT	ATGCGCCAGAAACGCATAGG
Cox57	TGGAAATGGAAGGCGGATTC	GGTGGAAGGCGTAAGCCTTT
Cox61	GAAGATAGAGCGGCAAGGAT	GGGATTTCAACTTCCGATAGA

**Table 3:** Results of *Coxiella burnetii* genotyping based on multi-spacer sequence typing (MST).

Sample ID	Sample Type	Cox 2	Cox 5	Cox 18	Cox 20	Cox 22	Cox 37	Cox 51	Cox 56	Cox 57	Cox 61	MST Genotype
MB92	Cattle Milk	3	2	6	1	5	10	4	10	6	5	61
MB98	Cattle Milk	3	8	1	6	5	4	5	4	6	11	62
AC3	Cattle Abortion	3	8	1	6	5	4	5	4	6	11	62
MG101	Goat Milk	3	8	1	6	5	4	5	4	6	11	62
AG4	Goat Abortion	3	8	1	6	5	4	5	4	6	11	62
MS45	Sheep Milk	3	8	1	6	5	4	5	4	6	11	62
MS51	Sheep Milk	3	8	1	6	5	4	5	4	6	11	62
AG1	Goat Abortion	3	11 *	1	6	5	4	5	4	6	11	63
AS3	Sheep Abortion	3	11 *	1	6	5	4	5	4	6	11	63
MB47	Cattle Milk	3	8	1	6	5	4	5	9	6	11	64
AC11	Cattle Abortion	3	8	1	6	5	4	5	9	6	11	64
AS25	Sheep Abortion	3	8	1	6	5	4	5	9	6	11	64
AS26	Sheep Abortion	3	8	1	6	5	4	5	9	6	11	64
MB14	Cattle Milk	3	8	1	6	5	4	5	15 ^¥^	6	11	65

* Novel allele for Cox 5; ¥ novel allele for Cox 56.

## Data Availability

Data that support the findings of this study are available within the article and from the corresponding author upon request.
